# CRISPR/Cas9-mediated disruption of *lipocalins, Ly6g5b*, and *Ly6g5c* causes male subfertility in mice

**DOI:** 10.1111/andr.13350

**Published:** 2022-12-10

**Authors:** Nobuyuki Sakurai, Yoshitaka Fujihara, Kiyonori Kobayashi, Masahito Ikawa

**Affiliations:** 1 Department of Bioscience and Genetics, National Cerebral and Cardiovascular Center, 6-1 Kishibeshinmachi, Suita, Osaka 564-8565, Japan; 2 Research Institute for Microbial Diseases, Osaka University, 3-1 Yamadaoka, Suita, Osaka 565-0871, Japan; 3 Graduate School of Frontier Biosciences, Osaka University, 3-1 Yamadaoka, Suita, Osaka 565-0871, Japan; 4 Graduate School of Pharmaceutical Sciences, Osaka University, 3-1 Yamadaoka, Suita, Osaka 565-0871, Japan; 5 Graduate School of Medicine, Osaka University, 3-1 Yamadaoka, Suita, Osaka 565-0871, Japan; 6 The Institute of Medical Science, The University of Tokyo, 4-6-1 Shirokanedai, Minato-ku, Tokyo 108-8639, Japan

**Keywords:** epididymis, knockout, Adam3, zona pellucida, sperm maturation

## Abstract

**Background:**

Spermatozoa become mature and competent for fertilization during transit from the caput epididymis to the cauda epididymis. However, detailed molecular mechanisms of epididymal sperm maturation are still unclear. Here, we focused on multiple epididymis-enriched genes: lipocalin family genes (*Lcn5, Lcn6, Lcn8, Lcn9*, and *Lcn10*) and Ly6 family genes (*Ly6g5b* and *Ly6g5c*). These genes are evolutionarily conserved in mammals and form clusters on Chromosomes 2 and 17 in the mouse, respectively.

**Objective:**

To clarify whether these genes are required for epididymal sperm maturation and acquisition of fertilizing ability, we generated knockout (KO) mice using the CRISPR/Cas9 system and analyzed their phenotype.

**Materials and methods:**

We generated four lines of KO mice: *Lcn9* single KO, the lipocalin family quadruple KO (*Lcn5, Lcn6, Lcn8*, and *Lcn10*), quintuple KO (*Lcn5, Lcn6, Lcn8, Lcn10*, and *Lcn9*), and double KO of Ly6 family genes (*Ly6g5b* and *Ly6g5c*).

**Results:**

While the *Lcn9* single KO did not affect male fertility, the quadruple KO and quintuple KO male mice were subfertile and mostly infertile, respectively, with a reduced amount of ADAM3, an essential protein for sperm binding to the zona pellucida. Further analysis revealed that the quintuple KO spermatozoa lack the CMTM2A/B that are required for ADAM3 maturation. Intriguingly, *Ly6g5b* and *Ly6g5c* double KO male mice also showed subfertility with reduced sperm ADAM3.

**Conclusion:**

These results suggest epididymal secretory proteins are involved in ADAM3 maturation and acquisition of sperm fertilizing ability.

## Introduction

Spermatozoa acquire their ability to fertilize and capability of forward motility during epididymal transit. This event is called epididymal sperm maturation. The epididymis comprises a dense convoluted duct that measures just over one meter in mice (see review by Hinton *et al*.^1^) and is divided into the caput, corpus, and cauda. Caput-spermatozoa have very low fertilizing potential although rates vary between species. However, most spermatozoa in the distal part of the epididymis can fertilize oocytes (see review by Bedford^2^), suggesting that some key factors controlling sperm maturation in the epididymis exist in the caput and/or corpus segment. Our laboratory has been searching genes predominantly expressed in the epididymis using a transcriptome database^3^ and generating knockout (KO) mice to investigate their physiological functions.^4–6^ In the present study, we focused on epididymis-enriched lipocalin and Ly6 family genes.

The lipocalin family consists of many small, diverse secreted proteins and is defined by a highly conserved three-dimensional structure.^7,8^ Most lipocalin family genes are evolutionarily conserved in humans and mice, forming clusters on chromosome 2 in mice.^9^ LCN2 is secreted in the female reproductive tract and binds to spermatozoa. Although *Lcn2* KO male mice are fully fertile, female mice show a significant reduction in pregnancy rate,^10,11^ suggesting that secreted LCN2 plays a key role in regulating sperm function in the female reproductive tract. Among the family, *Lcn5, Lcn6, Lcn8, Lcn9*, and *Lcn10* are predominantly expressed in the caput epididymis.^9^ KO mice for each of *Lcn6, Lcn8*, and *Lcn9* showed that these genes were individually dispensable for male reproduction.^12,13^ However, these studies did not explore the possibility that other lipocalin family members complimented the roles of the disrupted lipocalin genes.

The Ly6 family genes belong to the superfamily of lymphocyte antigen-6 (Ly6)/urokinase-type plasminogen activator receptor (uPAR) proteins. Most Ly6 superfamily members are evolutionarily conserved in humans and mice. Although the Ly6/uPAR family proteins share a common structure, their expression patterns and functions vary.^14^ Our group reported that KO male mice for *Ly6k, Lypd4*, and *Tex101*, members of the Ly6/uPAR family localized in testes and on spermatozoa, were infertile, respectively.^4,15,16^ A subset of the Ly6/uPAR family, called PATE, consists of secretory proteins and is primarily in the epididymis. The PATE family forms a cluster on chromosome 9, and fertility is reduced in male mice when most of the family is deleted.^4^ In this study, we focused on *Ly6g5b* and *Ly6g5c* as a member of the Ly6/uPAR family. They are adjacently located in the MHC class III region of mouse chromosome 17 and encode secretory proteins.^17^ However, no research has analyzed the roles of *Ly6g5b/c* genes *in vivo*.

The CRISPR/Cas9-mediated KO mouse generation and phenotype screening are a cost-effective and labor-effective approach to identify essential genes *in vivo* quickly.^18^ Using the CRISPR/Cas9 system, we can quickly induce indel mutation by introducing a gRNA/Cas9 complex into zygotes, but there is a risk that unexpected transcription products remain or are newly produced. Therefore, we applied a new approach introducing two gRNA-Cas9 complexes that delete the entire or most of the target gene.^5,19^ This study generated mice lacking Lipocalin family genes on chromosome 2 and *Ly6g5b/c* genes on chromosome 17 and investigated their roles in epididymal sperm maturation.

## Materials and Methods

### Animals

All mice used in this study were purchased from Japan SLC. Mice were acclimated to a 12-hour-light/12-hour-dark cycle. All animal experiments were approved by the Animal Care and Use Committee of the National Cerebral and Cardiovascular Center and the Research Institute for Microbial Diseases, Osaka University. Mice were maintained on the B6D2F1 mouse strain. All KO mouse lines generated in this study are available through RIKEN BRC (https://mus.brc.riken.jp/en/) or CARD (http://cardb.cc.kumamoto-u.ac.jp/transgenic/) bioresource centers.

### cDNA and RT-PCR

All tissues [brain, thymus, lung, heart, liver, spleen, kidney, testis, epididymis (caput, corpus, cauda), seminal vesicle, prostate, coagulation gland, ovary, and uterus] were collected from three C57BL/6NCr mice euthanized at 12 weeks old. These samples were homogenized in TRIzol (Ambion). Five μg of total RNA was reverse-transcribed to cDNA using SuperScript III First Standard Synthesis System, which contains 200 units of reverse transcriptase (Thermo Fisher Scientific), following the manufacturer’s instruction. Five ng of cDNA was used for PCR with primer sets (Table S1) and KOD DNA polymerase (KOD-FX Neo, TOYOBO). The amplification program was as follows: preincubation at 95°C for 5 min to activate the DNA polymerase, followed by 35 cycles of denaturation at 95°C for 30 s, annealing of the primers at 60°C for 30 s, and elongation at 72°C for 30 s.

### Egg collection, electroporation, and embryo transfer

CARD HyperOva (0.1 ml/mouse, Kyudo) was injected into the abdominal cavity of B6D2F1 females, followed by human chorionic gonadotropin (5 units, ASKA pharmaceutical) and natural mating with B6D2F1 males 48 hours after HyperOva injection. After 20 hours, we collected fertilized eggs with 2 pronuclei for genome editing. The crRNA and tracrRNA (Merck) were diluted with nuclease-free water (non-DEPC treated, Thermo Fisher Scientific). The mixture was denatured at 95°C for 1 minute and allowed to anneal by cooling gradually to room temperature. Each gRNA was mixed with the Cas9 protein solution (Thermo Fisher Scientific), and then incubated at 37°C for 5 minutes to prepare the gRNA/Cas9 ribonucleoproteins (RNPs) [final concentration: 50 ng/μl Cas9 for 20 ng/μl of each gRNA] (Table S2). The gRNA/Cas9 RNP solution was placed between the electrodes, and then the electroporation was done with the following conditions [resistance value: 550~600 Ω, poring pulse: 225 V (voltage), 2 ms (pulse amplitude), 50 ms (pulse interval), 4 (pulse number), 10% (attenuation), + (polarity), transfer pulse: 20 V (voltage), 50 ms (pulse amplitude), 50 ms (pulse interval), 5 (pulse number), 40% (attenuation), +/− (polarity)]. Electroporated embryos were cultured overnight, and more than 90% survived. Twenty embryos were transplanted into the oviducts of each pseudo-pregnant ICR recipient. After 19 days, offspring were obtained by natural birth or Caesarean section.

### Mating test

Eight-week-old KO male mice were caged with two B6D2F1 females per cage for 3 months. The number of delivered pups was counted, and they were removed immediately. Copulation was confirmed by checking for vaginal plugs every morning.

### Histological analysis of epididymis and sperm morphology

Epididymides were fixed in Bouin’s fluid (Polysciences) at 4°C overnight. Increasing ethanol concentrations and xylene-dehydrated fixed epididymides were then embedded with paraffin. Paraffin sections (5 μm) were stained with Mayer hematoxylin solution for 3 minutes, counterstained with eosin Y solution [53% ethanol, 0.3% eosin, and 0.5% acetic acid] for 3 minutes, dehydrated in increasing ethanol concentrations, and finally mounted in Entellan new (Merck). The cauda epididymal spermatozoa were observed with phase contrast microscopy.

### In vitro fertilization (IVF)

IVF using mouse spermatozoa was performed as described previously.^20^ Briefly, eggs collected from superovulated females 14 h after hCG injection were placed in the IVF medium (TYH or HTF). We used TYH for lipocalin quadruple KO and *Ly6g5b/c* KO, and switched to HTF for lipocalin quintuple KO because reagent supply became problematic during the COVID-19 situation. Epididymal sperm were collected from 3-month-old male mice and incubated in the IVF medium for 2 h for capacitation. Then, capacitated sperm were added to the medium containing eggs at a final concentration of 2 × 10^5^ sperm/ml and cultured overnight.

### Sperm-zona pellucida binding assay

Sperm-zona pellucida binding assay was performed with eggs from which the cumulus cells had been removed by treatment with bovine testicular hyaluronidase (175 U/ml, Merck) for 5 min. Cumulus-free eggs were placed in an IVF medium drop and inseminated. After 30 minutes of incubation, eggs were fixed with 0.25% glutaraldehyde. Spermatozoa bound to the zona pellucida were observed using an Olympus IX-73 microscope.

### Immunoblot

Immunoblot analysis was performed as described previously.^21^ Briefly, to collect testicular germ cells, seminiferous tubules were minced in PBS, and the suspension was filtered to remove spermatozoa and pieces of tissue. Sperm samples were collected from the cauda epididymis. We collected these samples from three males. The samples were homogenized in lysis buffer containing 1% Triton X-100 and 1% protease inhibitor (Nacalai Tesque) and then were centrifuged (10,000 g for 20 min at 4°C), and the supernatants were collected. Protein lysates were applied 20 μg per lane, separated by SDS/PAGE under reducing conditions, and transferred to PVDF membranes (Merck). Blocking was performed by incubation in 0.5% Tween20 and 10% skim milk in PBS for 90 min. After blocking, blots were incubated with primary antibodies diluted in blocking solution (for ADAM3; 1:1000; Santa Cruz; cat# sc-365288, for BASIGIN; 1:1000; Santa Cruz; cat# sc-46700, for OVCH2^22^; 1:500, for RNASE10; 1:100; LSBio; cat# LS-C296261, for GAPDH; 1:1000; Cell Signaling Technology; cat# 2118, for CMTM2A and CMTM2B^23^; 1:500, for IZUMO1^24^; 1:1000, for ADAM1B^25^; 1:500, for ADAM2; 1:500; Merck; cat# MAB19292) overnight at 4°C, and then incubated with secondary antibodies conjugated with horseradish-peroxidase (1:5000; anti-mouse secondary antibody; Proteinsimple; cat# 042-205, anti-rat secondary antibody; Jackson ImmunoResearch Laboratories; cat# 112-035-167, anti-rabbit secondary antibody; Jackson ImmunoResearch Laboratories; cat# 111-035-144). The detection was performed using Chemi-Lumi One Super (Nacalai Tesque).

### Statistical analysis

All values are shown as the mean ± SD of at least three independent experiments. Statistical analyses were performed using Student’s *t*-test inserted into Microsoft Excel after the data were tested for normality of distribution.

## Results

### Gene expression pattern

**Fig. 1.**
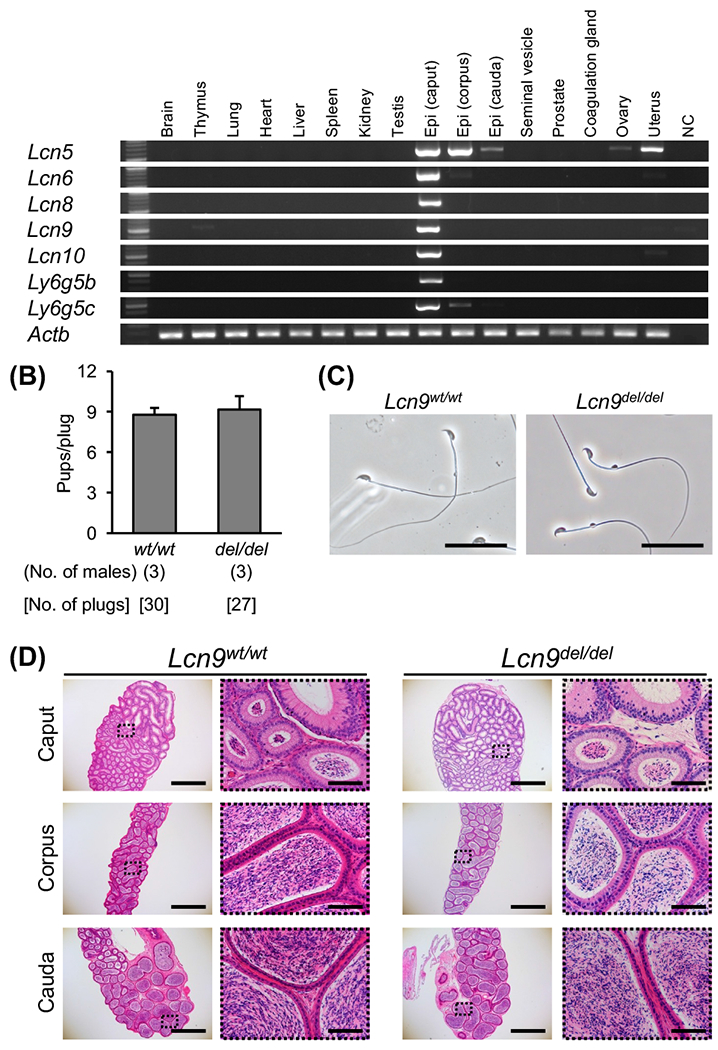
Multiple gene expression by RT-PCR and phenotype analysis of *Lcn9* KO mice. (A) Gene expression of *Lcn5, Lcn6, Lcn8, Lcn9, Lcn10, Ly6g5b*, and *Ly6g5c*. Actin β (*Actb*) was used as the control. Epididymis (Epi). (B) Male fertility. There was no difference in the average pups/plug ratio of WT and KO males (P = 0.68). (C) Cauda epididymal spermatozoa from WT B6D2F1 and *Lcn9*^*del/del*^ mice. Scale bars are 50 μm. (D) Histological analysis of the epididymis with H & E staining. The right panels are enlargements of the dashed areas in the left panels. Scale bars on the left and right panels are 1 mm and 100 μm, respectively.

RT-PCR analyzed gene expression patterns of genes we targeted in this study with multiple mouse tissues (Figure 1A). All the genes are predominantly expressed in the caput epididymis, while *Lcn5* is expressed in the epididymis and the uterus.

### Fertility of mice lacking lipocalin family genes

Lipocalin family genes analyzed here are located on the mouse chromosome 2qA3 locus. While *Lcn8, Lcn5, Lcn6*, and *Lcn10* form a cluster, *Lcn9* is approximately 137 kb away from it (see the gene information at Ensemble, URL: http://asia.ensembl.org/Mus_musculus/Location/View?g=ENSMUSG00000026937;r=2:25547964-25551989). Because there are other genes in between, we first generated *Lcn9* KO mice by introducing two gRNA/Cas9 protein complexes into pronuclear zygotes (*Lcn9*^*del/del*^, Supplementary Figure 1A) and analyzed male fertility. *Lcn9*^*del/del*^ males had fertility comparable to wild-type (WT) males, and there were no apparent problems with sperm morphology and epididymal histology (Figure 1B, C, and D).

**Fig. 2.**
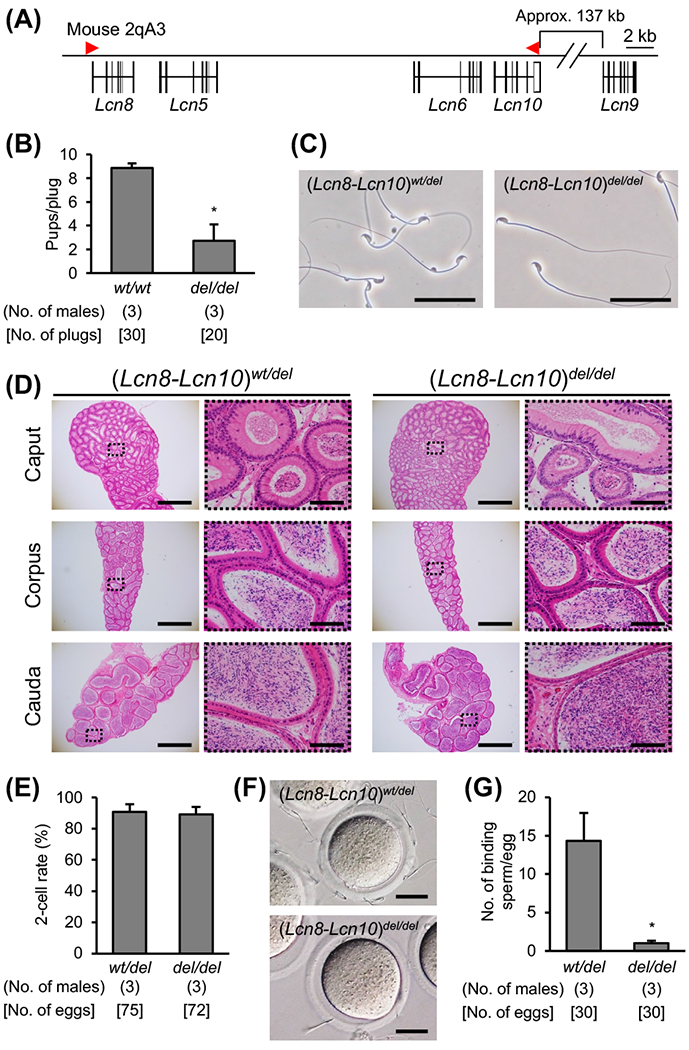
Phenotype analysis of *Lcn8-Lcn10* KO mice. (A) Genomic structure of *Lcn8, 5, 6, 10*, and *9*. Gene information was obtained from Ensemble (http://ensembl.org/index.html). (B) Number of pups per plug of WT female mice mated with KO male mice. WT B6D2F1 male mice were used as the control. * *P* < 0.01. (C) Cauda epididymal spermatozoa from (*Lcn8-Lcn10*)^*wt/del*^ and (*Lcn8-Lcn10*)^*del/del*^ mice. Scale bars are 50 μm. (D) Histological analysis of epididymis with H & E staining. The right panels are enlargements of the dashed areas in the left panels. Scale bars on the left and right panels are 1 mm and 100 μm, respectively. (E) *In vitro* fertilization ability analysis. (F and G) Sperm-zona pellucida binding assay. The average number of zona pellucida-binding spermatozoa obtained from heterozygote and homozygote mutant mice. Scale bars are 25 μm. * *P* < 0.01.

Next, we generated quadruple KO mice that deleted four lipocalin family genes, *Lcn8, Lcn5, Lcn6*, and *Lcn10* (Figure 2A and Supplementary Figure 1B). Control males consistently fathered litters with 9 pups per plug, and they generated approximately 30 plugs during the course of the mating trial. However, the *Lcn8-Lcn10* KO males fathered litters with less than 3 pups per plug, and the fertility was significantly lower than WT males (pups/plug ratio: 2.74 ± 1.37 in KO vs. 8.87 ± 0.38 in WT, *P* < 0.01, Figure 2B). There were no apparent defects in sperm morphology and epididymis histology of *Lcn8-Lcn10* KO mice (Figures 2C and D). When we performed IVF, the *Lcn8-Lcn10* KO spermatozoa efficiently fertilized cumulus-intact oocytes (Figure 2E) but barely bound to the zona pellucida when the spermatozoa were incubated with cumulus-free oocytes (Figures 2F and G).

**Fig. 3.**
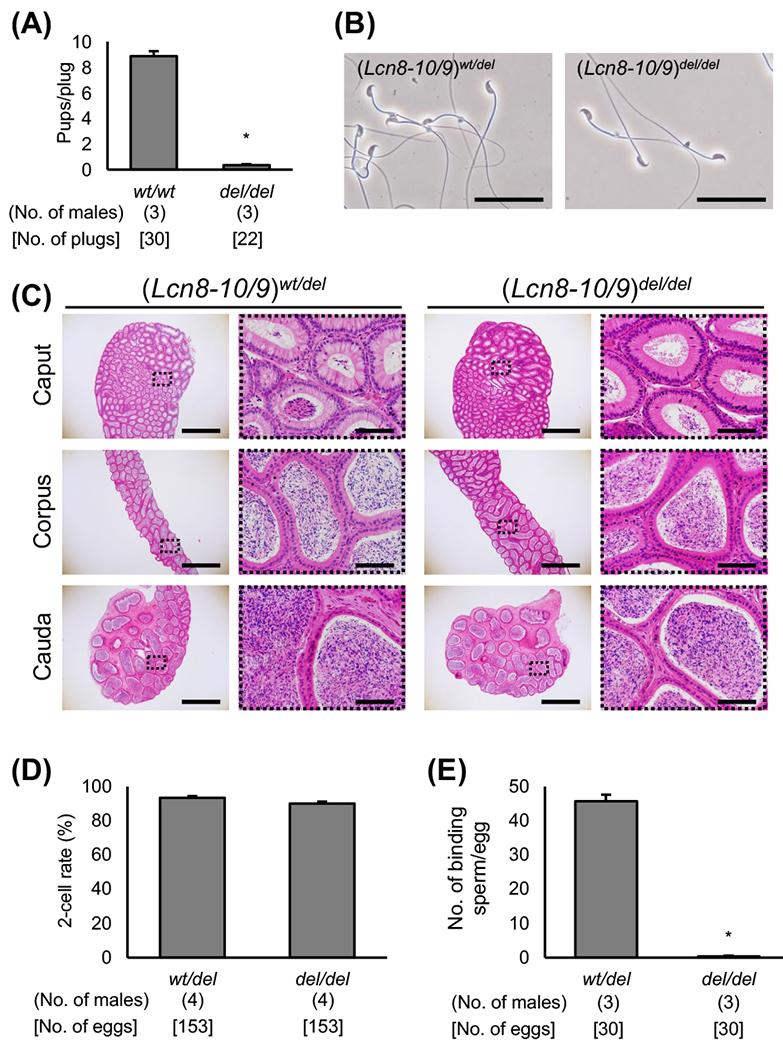
Phenotype analysis of *Lcn8-Lcn10* and *Lcn9* KO mice. (A) Value of pups per plug of WT female mice mated with KO male mice. WT B6D2F1 male mice were used as the control. * *P* < 0.01. (B) Cauda epididymal spermatozoa from (*Lcn8-10/9*)^*wt/del*^ and (*Lcn8-10/9*)^*del/del*^ mice. Scale bars are 50 μm. (C) Histological analysis of the epididymis with H & E staining. The right panels are enlargements of the dashed areas in the left panels. Scale bars on the left and right panels are 1 mm and 100 μm, respectively. (D) *In vitro* fertilization ability analysis. (E) Sperm-zona pellucida binding assay. Average number of zona pellucida-binding spermatozoa obtained from heterozygote and homozygote mutant mice. * *P* < 0.01.

Finally, we introduced two gRNA/Cas9 protein complexes to delete *Lcn9* in *Lcn8-10* KO zygotes and obtained quintuple KO mice. The *Lcn8-10/9* KO male fertility was significantly lower than that of mice mated with WT males (pups/plug ratio: 0.36 ± 0.06 vs. 8.87 ± 0.38, *P* < 0.01, Figure 3A). There were no apparent defects in sperm morphology and epididymal histology of *Lcn8-10/9* KO mice (Figures 3B and C). The quintuple KO sperm efficiently fertilized intact cumulus eggs *in vitro* (Figure 3D) but barely bound to the cumulus free zona pellucida (Figure 3E).

### Fertility of mice lacking Ly6g5b and Ly6g5c genes

**Fig. 4.**
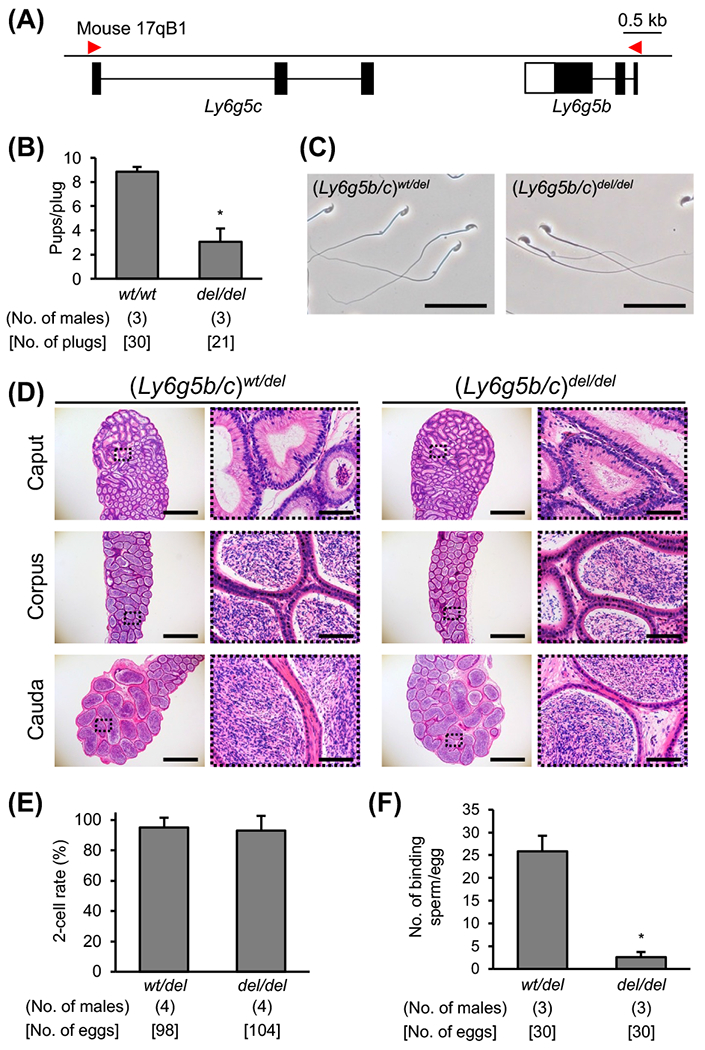
Phenotype analysis of *Ly6g5b/c* KO mice. (A) Genomic structure of *Ly6g5c* and *b*. Gene information was obtained from Ensemble. (B) Value of pups per plug of WT female mice mated with KO male mice. WT B6D2F1 male mice were used as the control. * *P* < 0.01. (C) Cauda epididymal spermatozoa from (*Ly6g5b/c*)^*wt/del*^ and (*Ly6g5b/c*)^*del/del*^ mice. Scale bars are 50 μm. (D) Histological analysis of the epididymis with H & E staining. The right panels are enlargements of the dashed areas in the left panels. Scale bars on the left and right panels are 1 mm and 100 μm, respectively. (E) *In vitro* fertilization ability analysis. (F) Sperm-zona pellucida binding assay. The average number of zona pellucida-binding spermatozoa obtained from heterozygote and homozygote mutant mice. * *P* < 0.01.

*Ly6g5b* and *Ly6g5c* are located on mouse chromosome 17qB1 locus (see the gene information at Ensemble, URL: https://asia.ensembl.org/Mus_musculus/Location/View?db=core;g=ENSMUSG00000043807;r=17:35332924-35334404). We generated *Ly6g5b* and *Ly6g5c* KO mice using the same method (Figure 4A and Supplementary Figure 1C). The *Ly6g5b/c* KO male fertility was significantly lower than that of WT males (pups/plug ratio: 3.07 ± 1.08 vs. 8.87 ± 0.38, *P* < 0.01, Figure 4B). There was no difference in sperm morphology and epididymal histology in *Ly6g5b/c* KO mice (Figure 4C and D). The *Ly6g5b/c* KO sperm efficiently fertilized intact cumulus eggs *in vitro* (Figure 4E). However, in the sperm binding assay, *Ly6g5b/c* KO spermatozoa barely bound to the zona pellucida when the spermatozoa were incubated with cumulus-free oocytes (Figure 4F).

### Immunoblot analysis for ADAM3-related proteins

**Fig. 5.**
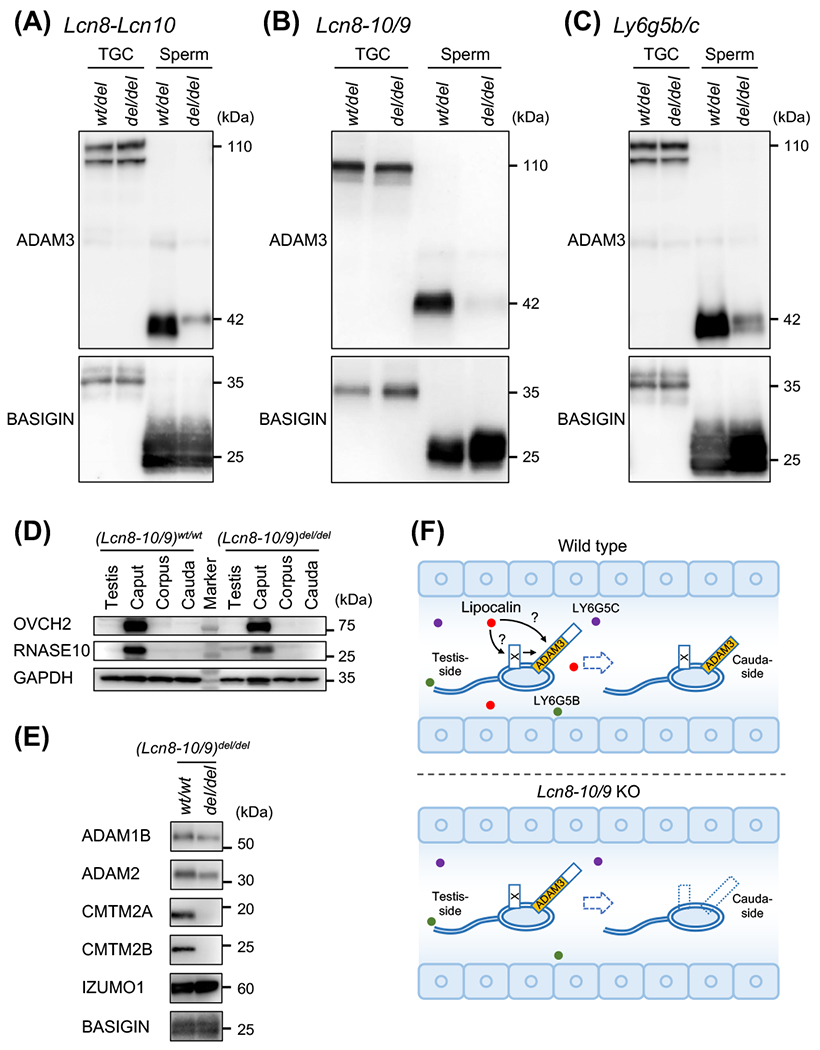
Immunoblot analysis. (A-C) Immunoblot analysis for ADAM3. The study was performed using testicular germ cells (TGC) and cauda epididymal spermatozoa obtained from (A) *Lcn8-Lcn10*, (B) *Lcn8-10/9*, and (C) *Ly6g5b/c* mutant mice. BASIGIN was used as the control. (D) Immunoblot analysis for OVCH2 and RNASE10 using protein lysates collected from WT and *Lcn8-10/9* KO testis, caput, corpus, and cauda sperm. GAPDH was used as the control. (E) Immunoblot analysis for ADAM1B, ADAM2, CMTM2A, CMTM2B, and IZUMO1 using cauda sperm lysates collected from WT and *Lcn8-10/9* KO mice. BASIGIN was used as the control. (F) Schematic view of this study.

*Adam3* KO spermatozoa can fertilize intact cumulus eggs but cannot bind to the zona pellucida,^26,27^ similar to the phenotype we found in our KO mice. Because of this similarity, we analyzed the ADAM3 protein levels by immunoblot analysis. ADAM3 protein exists on the sperm surface, and a 110 kDa precursor form of ADAM3 is processed into the 42 kDa mature protein during the transition from the testis to the epididymis.^28^ We used BASIGIN as a control because BASIGIN also localizes on the spermatozoa and is processed from 37 kDa to 26 kDa during the transfer from the testis to the epididymis.^29^ There was no apparent difference in ADAM3 levels in WT and KO testicular germ cell (TGC) samples (Figure 5). However, in cauda epididymal sperm samples derived from *Lcn8-Lcn10* KO males, the ADAM3 signal was weaker than that of heterozygous mutant males (Figure 5A). In *Lcn8-10/9* KO spermatozoa, the ADAM3 signal nearly disappeared (Figure 5B). In *Ly6g5b/c* mutants, the signal was also weak in homozygous mutant males (Figure 5C).

During epididymal sperm transition, Ovochymase 2 (OVCH2), and RNASE10 are secreted from the caput epididymis and are involved in ADAM3 processing.^22,30^ Therefore, we first examined them by immunoblot analysis, but we did not see any significant differences between WT and *Lcn8-10/9* KO samples (Figure 5D). Finally, we examined sperm surface proteins, ADAM2 and CMTM2A/B, which are essential for ADAM3 maturation.^23^ There were no differences in ADAM2, but CMTM2A/B disappeared from *Lcn8-10/9* KO spermatozoa. (Figure 5E).

## Discussion

In mammals, immature spermatozoa generated in the testes must pass through the epididymis to gain their ability to fertilize.^31^ By knocking out genes in mice, we have searched for genes that are expressed in the epididymis and are involved in epididymal sperm maturation.^4,5,22^ Some epididymis-enriched genes are remarkably homologous to each other and are expected to play redundant roles; thus, the single KO approach is not a feasible way to study these genes. In this study, we took advantage of the CRISPR/Cas9 system by knocking out the epididymis-enriched *Lcn8/5/6/10/9* and *Ly6g5b/c* gene clusters and found infertility phenotypes. It is important to remember that deletions of gene regions can potentially delete unidentified protein-coding regions, delete non-coding RNAs, or affect the expression of neighbor genes. Although the transgenic rescue approach is ideal for proving if the targeted gene is responsible for the phenotype, it is also difficult to choose which gene to express when we disrupt multiple genes. In this study, instead of adding a transgene, the additional defect observed with *Lcn9* single KO underpins that *Lcn9* codes protein that complements LCN protein functions.

As we did not see any overt morphological abnormalities and detected a normal amount of proteins involved in ADAM3 maturation, OVCH2 and RNASE10, in *Lcn8-10/9* KO mice, lipocalins are not potentially critical for epididymal development per se. Instead, we found that sperm plasma membrane proteins CMTM2A/B disappeared from the *Lcn8-10/9* KO spermatozoa suggesting that lipocalins affect ADAM3 localization through CMTM2A/B on the sperm heads. However, because the mechanisms of how CMTM2A/B regulates ADAM3 is unknown, further studies are needed to elucidate entire molecule mechanisms (Figure 5F). Furthermore, as secreted protein LCN6 binds to sperm heads and tails in humans,^32^ sperm affinity of lipocalins is also the key to understanding their molecular function.

Our study suggests that *Ly6g5b/c* genes are also involved in the ADAM3 maturation process in the epididymis. It should be noted that previous studies proved several Ly6 family proteins (e.g., LY6K, LYPD4, and TEX101) are individually required for sperm ADAM3 processing and male fertility.^33^ Therefore, it is not always true to consider family genes complementary. Intriguingly, Ly6 family proteins are expressed in various male reproductive organs (testis, spermatozoa, and epididymis). A transgenic approach with different promoters and/or swapping coding sequences by CRISPR/Cas9 mediated knockin might be helpful to assess individual functions *in vivo*. There still remain many proteins coded by gene clusters that seem to control epididymal sperm maturation.^4,22^ Our approach of deleting gene clusters will be helpful in deciphering the functions for epididymal sperm maturation and male fertility.

## Supplementary Material

Sup1 Suppl. Fig. 1. Strategy for (A)*Lcn9* (B) *Lcn8-Lcn10*, and (C) *Ly6g5b/c* KO mice generation. Gene information was obtained from Ensemble.

TableS1

TableS2
