# Supplementary figures and images for: CRISPR/Cas9-mediated disruption of lipocalins, Ly6g5b, and Ly6g5c causes male subfertility in mice
Source: Andrology. Author manuscript; Available in PMC 2024 Jul 1. (PMC10506895; doi:10.1111/andr.13350)

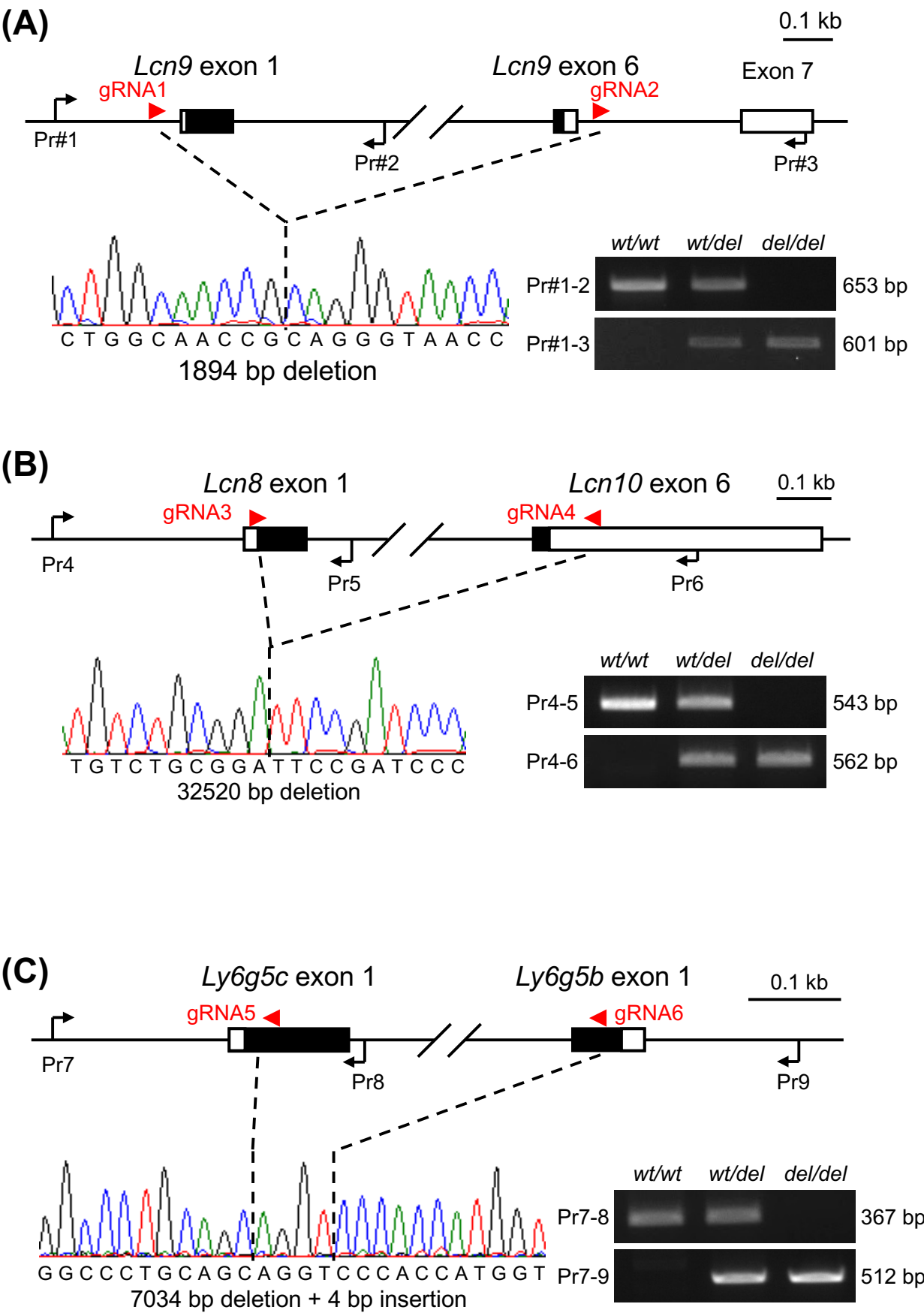

Supplement: Sup1 — Suppl. Fig. 1. Strategy for (A)Lcn9 (B) Lcn8-Lcn10, and (C) Ly6g5b/c KO mice generation. Gene information was obtained from Ensemble. [file NIHMS1925039-supplement-Sup1.pdf]
