# Supplementary material for: CRISPR/Cas9-mediated disruption of lipocalins, Ly6g5b, and Ly6g5c causes male subfertility in mice
Source: Andrology. Author manuscript; Available in PMC 2024 Jul 1. (PMC10506895; doi:10.1111/andr.13350)
Supplement: TableS1 [file NIHMS1925039-supplement-TableS1.docx]

Supplemental Table S1. Primer sequences for the multiple tissue expression analysis.

| **Genes** | **Forward (5’-3’)** | **Reverse (5’-3’)** | **Total cycles** | **Predicted size**  **(bp)** | **Ensembl**  **Accession #** |
| --- | --- | --- | --- | --- | --- |
| *Lcn5* | atgtgctcagttgctaggca | gatctgtcccgattgcaatgc | 35 | 576 | ENSMUSG00000026937 |
| *Lcn6* | atgaaggtgatccttctgac | ctggaggatcttgtgtgcacaagtg | 35 | 623 | ENSMUSG00000045684 |
| *Lcn8* | atggaagctaggctgctgag | gaccagtccctctttcaagagctcag | 35 | 525 | ENSMUSG00000036449 |
| *Lcn9* | atggtactactactagtcct | taaaaagcaaaaatctttttttg | 35 | 537 | ENSMUSG00000023210 |
| *Lcn10* | atgaagttagagatggccct | aggcaggatcgtatgtgcacagg | 35 | 546 | ENSMUSG00000047356 |
| *Ly6g5b* | atggtgatcaccatctataa | gggtctatcccagggaaggacc | 35 | 417 | ENSMUSG00000043807 |
| *Ly6g5c* | atgctttttatggcaggccc | gtgcatgctattctttctgttgtcc | 35 | 442 | ENSMUSG00000034482 |
| *Actb* | catccgtaaagacctctatgccaac | atggagccaccgatccaca | 35 | 171 | ENSMUSG00000029580 |
