# Supplementary material for: CRISPR/Cas9-mediated disruption of lipocalins, Ly6g5b, and Ly6g5c causes male subfertility in mice
Source: Andrology. Author manuscript; Available in PMC 2024 Jul 1. (PMC10506895; doi:10.1111/andr.13350)
Supplement: TableS2 [file NIHMS1925039-supplement-TableS2.docx]

Supplemental Table S2. Primer sequences for the genotyping and gRNA sequences.

| **Genes** | **gRNA (5’-3’)** | **Forward (5’-3’)** | **Reverse (5’-3’)** | **Total cycles** | **Predicted size(bp)** | **Ensembl**  **Accession #** |
| --- | --- | --- | --- | --- | --- | --- |
| *Lcn9* | ctggcaaccgcagaacagaa (gRNA1) | ccacatgatctcagcaggct  (Pr-1) | aaggaaaggctgagtggtgg  (Pr-2) | 40 | 653 | ENSMUSG00000023210 |
|  | ggggtgttgggaaccgggca (gRNA2) | – | cggagatatgaccacaggcc  (Pr-3) | 40 | 601 |  |
| *Lcn8* | caatgtctgcggattcttcc (gRNA3) | agactcaggcctccttgttc  (Pr-4) | ggttgtacactctgctccct  (Pr-5) | 40 | 543 | ENSMUSG00000036449 |
| *Lcn10* | tctgggatcggaagatgcaa (gRNA4) | – | aggaaatgggagcagagtga  (Pr-6) | 40 | 562 | ENSMUSG00000047356 |
| *Ly6g5c* | acaaggcttgggggacgcca (gRNA5) | cccggttgcttttgtcagag  (Pr-7) | agccacttaccgagaaccaa  (Pr-8) | 40 | 367 | ENSMUSG00000034482 |
| *Ly6g5b* | accatggtgggctttgcgat (gRNA6) | – | ccagacaatgtgggtgagga  (Pr-9) | 40 | 512 | ENSMUSG00000043807 |
